# Efficacy and safety of astragalus injection combined with Western medicine in the treatment of early diabetic nephropathy: A protocol for systematic review and meta-analysis

**DOI:** 10.1097/MD.0000000000025096

**Published:** 2021-03-26

**Authors:** Guojing Li, Bichen Ai, Weihua Zhang, Xingzhong Feng, Min Jiang

**Affiliations:** a Department of TCM, Capital Medical University Affiliated Beijing Shijitan Hospital, Beijing; b College of Chinese medicine, Hunan University of Chinese Medicine, Changsha, Hunan Province; c Yuquan Hospital of Tsinghua University, Beijing, China.

**Keywords:** astragalus injection, diabetic nephropathy, meta-analysis, protocol, western medicine treatment

## Abstract

**Background:**

Diabetic nephropathy (DN) is the most common microvascular complication of diabetes. Its clinical manifestation is proteinuria, and it is a common cause of renal failure. At present, angiotensin converting enzyme inhibitors (ACEI) and angiotensin II receptor antagonists are often used to treat early DN, and they have good curative effect. On this basis, the treatment of early DN with the combination of astragalus injection is becoming more and more widespread. Therefore, the purpose of this study is to prove the efficacy and safety of astragalus injection combined with Western medicine in the treatment of early DN, and to provide reference value for clinical practice in the future.

**Methods:**

English databases (PubMed, Embase, Web of Science, the Cochrane Library) and Chinese databases (China National Knowledge Infrastructur, Wanfang, VP Information Chinese Journal Service Platform, China Biology Medicine disc) will be searched by computer. From the establishment of the database to February 2021, a randomized controlled trial of astragalus injection combined with Western medicine in the treatment of early DN will be conducted. Two researchers independently evaluate the quality of the included study and extract the data. Included literature is analyzed by Meta with RevMan5.3 software.

**Results:**

In this study, the efficacy and safety of astragalus injection combined with Western medicine in the treatment of early DN are evaluated by serological indexes such as Urinary albumin excretion rates (UAER), serum creatinine and blood urea nitrogen, as well as the adverse reactions of drugs.

**Conclusion:**

This study will provide reliable evidence-based evidence for astragalus injection combined with Western medicine for the treatment of early DN.

**OSF Registration number:**

DOI 10.17605/OSF.IO/A9JGP

## 1 Introduction

Diabetic nephropathy (DN) refers to glomerulosclerosis caused by diabetes, which is one of the complications of diabetic systemic microangiopathy. In the early stage of DN [urinary albumin excretion rates (UAER) is 20-200251658240 μg min^−1^], if there is no timely intervention, the UAER value will increase year by year, and eventually renal failure may occur. Renal failure caused by DN accounts for 13.3% of all dialysis patients.^[1]^ Therefore, the timely use of drugs for early treatment is of great significance.^[2]^

A large number of studies^[3]^ believe that reducing urinary protein can delay the progression of DN. And commonly used drugs in clinic are angiotensin converting enzyme inhibitors (ACEI) and angiotensin II receptor antagonists, which have good effects.^[4]^ At present, Astragalus injection combined with Western medicine is widely used in clinical, which has more advantages than using Western medicine alone. The results of several randomized controlled trials show that astragalus injection can reduce urinary protein excretion and improve glucose and lipid metabolism in patients with DN to a certain extent.^[5–8]^ This study included domestic and foreign randomized controlled trials of astragalus injection combined with Western medicine for the treatment of early DN, objectively evaluated its efficacy and safety, and provided an effective basis for clinical promotion.

## 2 Methods

### 2.1 Protocol register

This protocol of systematic review and meta-analysis has been drafted under the guidance of the preferred reporting items for systematic reviews and meta-analyses. In addition, it has been registered on open science framework (OSF) (Registration number: DOI 10.17605/OSF.IO/A9JGP).

### 2.2 Ethics

This study does not involve the privacy of the patients and does not require the approval of the ethics committee and informed consent of the patients.

### 2.3 Eligibility criteria

#### 2.3.1 Types of studies

We will collect all available randomized controlled trails on astragalus injection combined with Western medicine for the treatment of early DN, unlimited magazine, publication time, and whether to use the blind method. The language is limited to Chinese and English.

#### 2.3.2 Research object

The subjects are diagnosed as diabetic patients, who meet the diagnostic criteria of WHO diabetes in 1999.^[9]^ According to the staging criteria of DN of Mogensen,^[10]^ they are diagnosed as early DN (DN stage III), that is, microalbuminuria. The urine albumin of UAER is 30 to 300 mg at 20 to 200 or 24 hours.

#### 2.3.3 Intervention measures

Control Group: ACEI or angiotensin II receptor blocker + routine therapy (exercise, diet, hypoglycemic, hypotensive, lipid-regulating, etc.).

Treatment Group: Astragalus injection (dosage form, dosage, and usage are not limited) is added on the basis of treatment in the control group.

#### 2.3.4 Outcome index

The main indicators are urinary albumin excretion rate (UAER). The secondary indicators are serum creatinine, blood urea nitrogen, glomerular filtration rate, glycosylated hemoglobin, and adverse drug reactions.

### 2.4 Exclusion criteria

1. Renal damage caused by other causes.
2. The treatment measures are not consistent with each other.
3. The literature of outcome index can not be obtained.
4. The research in which the full text is not available.
5. The data is wrong or the information is incomplete, but the research that can not be solved by the author can not be solved.
6. For the repeatedly published literature, select the literature with the most comprehensive data and the highest quality.

### 2.5 Retrieval strategy

The search uses the combination of subject words and free words to search China National Knowledge Infrastructur, Wanfang Database, VP Information Chinese Journal Service Platform, and China Biology Medicine disc with the Chinese words of “Astragalus injection” and “Diabetic Nephropathy,” and in the English database, including PubMed, Embase, Web of Science, and the Cochrane Library, the English search words are “Astragalus Injection” and “diabetic nephropathy”. The search time is from the establishment of the database in February 2021, and randomized controlled trials of astragalus injection combined with Western medicine for the treatment of early DN are collected. Take PubMed as an example, the retrieval strategy is shown in Table 1.

**Table 1:** PubMed database retrieval strategy.

Number	Search terms
#1	Astragalus propinquus[MeSH]
#2	Astragalus[Title/Abstract]
#3	Astragalus injection[Title/Abstract]
#4	Astragalus membranaceus [Title/Abstract]
#5	#1 OR #2 OR #3OR #4
#6	Diabetic nephropathies[MeSH]
#7	diabetic nephropathy[Title/Abstract]
#8	#6 OR #7
#9	#5 AND #8

### 2.6 Data screening and extraction

**Figure 1:**
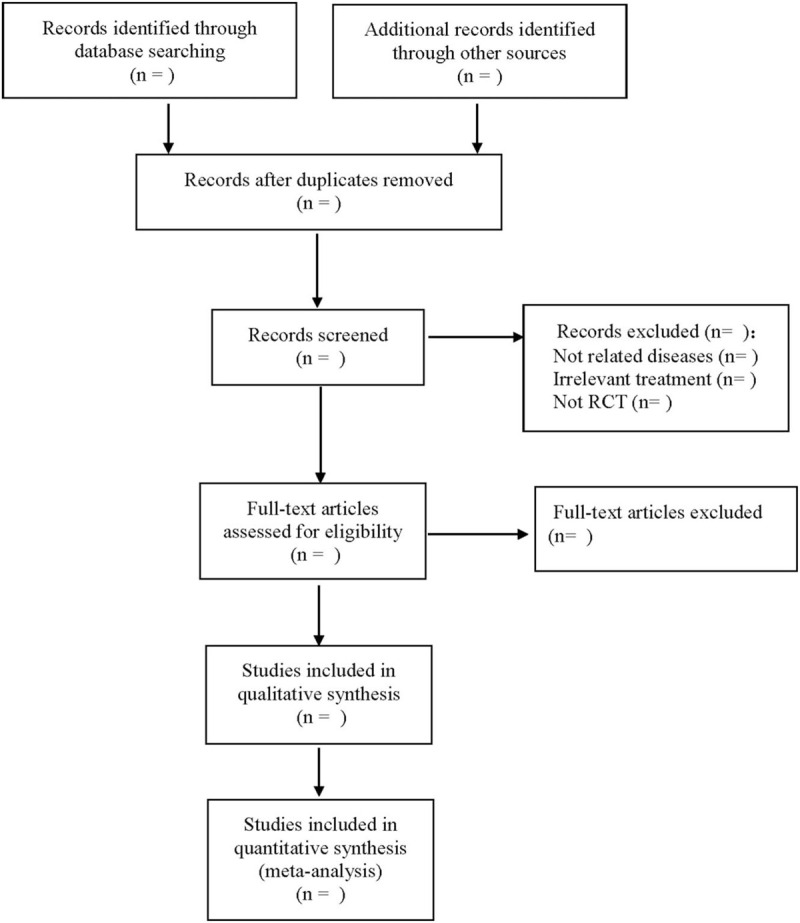
The process of literature filtering.

With reference to the method of screening the literature in the Cochrane Collaboring Network System Reviews Manual 5.0, the 2 researchers use EndNote X7 document management software to download the literature, remove the duplicate literature, and then read the full text to further screen the literature according to the title and abstract. Finally, the required literature is included. The 2 researchers independently screen and extract the literature information, and check with each other. If they encounter differences, they will negotiate and resolve them or ask the third researcher to assist in judgment. The extraction of the data mainly includes the basic information included in the literature, including the title, the name of the first author, the year of publication, the magazine published and the country in which the study is carried out, the basic information of the subjects, including average age, sex, number of samples, race, severity, etc.; the intervention methods of the treatment group and the control group, including the dose of astragalus injection, the type and usage of Western medicine, etc.; the relevant information of the outcome index. Information about literature quality evaluation includes random grouping method; allocation hiding method; blind method and so on. The flowchart is used to show the research selection process (Fig. 1).

### 2.7 Literature quality evaluation

The Cochrane Collaboration's Tool for assessing risk of bias is used in Review Manager 5.3 software to estimate risk of bias. The evaluation content includes the following 7 parts: ① generation of random sequence; ② allocation concealment of random schemes; ③ blind method was carried out by the subjects and the implementers of the intervention; ④ results the evaluators were blinded; ⑤ integrity of outcome index data; ⑥ selective reporting; ⑦ other aspects of bias. Two researchers will give low risk, unclear, and high risk judgments on the above items, and cross-check them respectively. If there is any disagreement, it shall be discussed. If no agreement can be reached, it shall be negotiated with the researcher of the third party.

### 2.8 Statistical analysis

RevMan5.3 software is used for meta-analysis. The dichotomous variable is expressed by the relative ratio. For measurement data, if the measurement tool and measurement unit are consistent, use the weighted mean difference to express, if the measurement tool or measurement unit is inconsistent, then use the standard mean difference as the effective quantity. The heterogeneity between the results is analyzed by *χ*^2^ tests, the test level α = 0.05, combined with *I*^2^ to judge the heterogeneity, if *P≥*. 1, *I*^*2*^*≤*50%, the heterogeneity is low, and the fixed effect model is used. If *P* < . 1, *I*^*2*^ < 50%, the source of heterogeneity should be analyzed because there is obvious heterogeneity between studies. The source of heterogeneity should be analyzed. If there is no obvious clinical and methodological heterogeneity, random effect model is used for analysis; if there is obvious clinical heterogeneity and methodological heterogeneity, methods such as subgroup analysis or sensitivity analysis are used. If the clinical heterogeneity is too obvious, it is impossible to carry out subgroup analysis, only descriptive analysis.

#### 2.8.1 Dealing with missing data

If there is missing data in the article, contact the author through email to the relevant information. If the author cannot be contacted, or if the author has lost the relevant data, a descriptive analysis is performed, not a meta-analysis.

#### 2.8.2 Subgroup analysis

According to the different types of Western medicine in the treatment group, the subgroup analysis is carried out; according to the age of the patients, the patients can be divided into 3 subgroups: the young, the middle-aged and the elderly; the subgroup analysis is carried out according to the course of treatment.

#### 2.8.3 Sensitivity analysis

In order to judge the stability of the outcome index, sensitivity analysis is used to analyze each outcome index.

#### 2.8.4 Assessment of reporting biases

If the number of studies included in an outcome indicator is not less than 10, a funnel chart is used to assess publication bias. In addition, Egger and Begg test are used for the evaluation of potential publication bias.

## 3 Discussion

DN is one of the serious complications of diabetes, and it is a common renal disease in the elderly, which can accumulate renal vessels, glomerulus, renal tubules, and renal interstitium. Its clinical manifestations are proteinuria, edema, hypertension, and progressive damage of renal function. The deterioration of the disease developed into uremia. Clinical commonly used drugs are ACEI and angiotensin II receptor blocker to reduce urinary protein, which has a good effect.^[4]^

According to the theory of traditional Chinese medicine, most of DN is the type of deficiency of both Qi and Yin with blood stasis, deficiency of Qi leads to weak blood flow, and blood stasis is formed over time, which affects the movement of Qi and blood.^[11]^ Astragalus injection is made of Astragalus extract, which has the effect of tonifying the spleen and stomach, tonifying Qi and rising Yang, promoting Qi and diuresis.^[12]^ Modern pharmacological studies have shown that oral administration of Astragalus membranaceus decoction can enhance the phagocytosis of reticuloendothelial system, promote metabolism, dilate coronary artery, renal vessels and systemic peripheral vessels, and diuretic effect is equivalent to dihydrochlorothiazide 0.2 mg/ kg, and the diuretic effect lasts for a long time.^[13,14]^ The results show that astragalus injection can improve urinary albumin excretion, reduce excessively high glomerular filtration rate, and lower blood glucose and triglyceride in patients with early DN, indicating that astragalus injection has a protective effect on the kidney of patients with DN and can improve glucose and lipid metabolism.^[15,16]^ The mechanism of Astragalus injection may be related to its inhibition of TGF- β in renal cortex and over expression of genes, so as to play the role of anti-fibrosis and effectively protect the kidney.^[17,18]^

In recent years, the study^[19–23]^ found that Astragalus injection combined with Western medicine was effective in the treatment of early DN. In most experiments, astragalus injection combined with ACEI and angiotensin II receptor antagonist were used to improve the symptoms of early DN, especially in improving glucose and lipid metabolism. Therefore, it is necessary to systematically evaluate and meta analyze the efficacy of astragalus injection combined with Western medicine for the treatment of early DN, and objectively evaluate its efficacy, so as to provide an alternative treatment for clinical treatment of early DN. However, this study also has some limitations. This article only involves Chinese and English literature, and the relevant studies in other languages will be ignored. At the same time, due to the different use of Western medicine in different research institutes, there is a certain clinical heterogeneity between the studies. More high-quality studies are needed to confirm the efficacy of astragalus injection combined with Western medicine for the treatment of early DN.

## Author contributions

**Data collection:** Guojing Li and Bichen Ai.

**Funding support:** Min Jiang.

**Literature retrieval:** Guojing Li and Bichen Ai.

**Software operating:** Weihua Zhang and Xingzhong Feng.

**Supervision:** Min Jiang.

**Writing – original draft:** Guojing Li and Bichen Ai.

**Writing – review & editing:** Guojing Li and Min Jiang.
